# Permittivity-Based Water Content Calibration Measurement in Wood-Based Cultural Heritage: A Preliminary Study

**DOI:** 10.3390/s22062148

**Published:** 2022-03-10

**Authors:** Livio D’Alvia, Emanuele Piuzzi, Andrea Cataldo, Zaccaria Del Prete

**Affiliations:** 1 Department of Mechanical and Aerospace Engineering, Sapienza University of Rome, 00184 Rome, Italy; zaccaria.delprete@uniroma1.it; 2 Department of Information Engineering, Electronics and Telecommunications, Sapienza University of Rome, 00184 Rome, Italy; emanuele.piuzzi@uniroma1.it; 3 Department of Engineering for Innovation, University of Salento, 73100 Lecce, Italy; andrea.cataldo@unisalento.it

**Keywords:** calibration procedure, water content measurement, dielectric permittivity measurement, cultural heritage

## Abstract

In this work, the dielectric permittivity of four kinds of wood (Fir, Poplar, Oak, and Beech Tree), used in Italian Artworks and structures, was characterized at different humidity levels. Measurements were carried out using three different probes connected to a bench vector network analyzer: a standard WR90 X-band waveguide, a WR430 waveguide, and an open-ended coaxial probe. In particular, we investigated the dispersion model for the four wood species, showing how a log-fit model of the open-ended data presents a determination coefficient *R^2^* > 0.990 in the 1–12 GHz frequency range. This result has proven helpful to fill the frequency gap between the measurements obtained at different water contents with the two waveguide probes showing an *R^2^* > 0.93. Furthermore, correlating the log-fit vertical shift with the water content, it was possible to find a calibration curve with a linear characteristic. These experimental results will be helpful for on-site non-invasive water monitoring of wooden artworks or structures. Moreover, the final results show how the open-ended coaxial probe, with a measurement deviation lower than 7% from the waveguide measurements, may be used directly as a non-invasive sensor for on-site measurements.

## 1. Introduction

Water content is undoubtedly one of the most critical parameters in artworks, historical objects, and buildings conservation [1,2], being the promoter of changes in the size and shape, and accelerating the deterioration rate due to chemical reactions and biological deterioration sources. In particular, the physical–mechanical properties of wood objects and structures are more affected by water content variations, due to the highly hygroscopic nature of the material, than other materials commonly used in historic buildings (stones, bricks). In the study of moisturized wood, it must be taken into account that two particular points characterize the phenomenon of water adsorption: in the range 0% to 25% or Fiber Saturation Point (FSP), the water is transferred into the wood cell wall; above this point, the cell walls are fully saturated, and the water passes into cavity cells [3,4]. A gravimetric water content ~140% represents the Water Saturation Point (WSP). Below FSP, changes in the water content affect the physical-mechanical and rheological properties of wood (swelling, shrinking, modulus of rigidity or elasticity, or strength values), while above 5% of water content risks for insect infestation increase.

If gravimetric methods are the most accurate procedures to evaluate water content in the objects, they are destructive and require taking samples of the object under investigation. For these reasons, the curators’ attention in monitoring historical objects through non-invasive and ad-hoc instrumentations, devices, and methodologies has considerably increased in the last ten years. As a result, a non-homogeneous variety of methods and measurement instrumentation exist to assess the water level, which can be classified according to the physical principle interested [5,6]. From the literature, it appears that methods that associate the water level to the electrical properties of materials represent attractive solutions because they are very sensitive to moisture [7]. Therefore, a brief description of the common techniques and instruments is proposed below [8].

Electrical conductivity-based methods are micro-destructive methods based on measuring the current intensity, at the discrete current domain, which flows through two or four probes either in direct contact with the surface layer or implanted in the object at the maximum depth of 10 cm. In wood-based objects, the resistance decreases with increased water content. For this method, sources of error are represented by dissolved salts, temperature gradient around the measurement points, or discontinuities inside the wood-based object. At last, the principal limitation for this method regards the accuracy of 1.0% limited in the range 6% to the FSP point; above and below this range the accuracy decreases significantly. This differentiates from the microwave method that permits measuring water content in the range of 0%–FSP without losing accuracy [9].

Electrical capacity methods are characterized by contact probes that measure the dielectric variation produced by an increase/decrease in water content between the probes armor in terms of capacitance at low frequencies. At the base of its operation, there is the variation in relative dielectric permittivity *ε_r_* due to the presence of water; in fact, a dry sample presents a permittivity between two and eight, and it changes proportionally with the water variation (water *ε_r_* = 80). For this method, sources of error are represented by variation of density, cavity, or internal discontinuities. This method presents an acceptable accuracy in the range of 3% to the FSP point [10]. Like the microwave methods, the capacitive one permits correlating the water content with dielectric permittivity but at only one frequency for commercial devices, or limited to 100 kHz to 10 MHz for customized one with an accuracy of 1.0% [11].

Waveguides, opened-ended probes (OEPC), or planar resonators characterize the microwave method. The probes emit energy inside the object under investigation at higher frequencies (0.3–300 GHz) than capacitive ones. They are based on the measure of perturbation of the electromagnetic field due to the interaction with the water molecules [12]. For this method, sources of error are represented by temperature, material density, and thickness [13]. Due to the waveguide high-cost to accuracy ratio, measurements are generally limited to methodological/calibration concepts. Nevertheless, the rapid growth of low-cost open or resonant probes has allowed us to apply this method in the field without losing accuracy [14].

A particular microwave method is the Time-domain Reflectometry (TDR), which measures the elapsed time between transmission and response of a radio signal to evaluate the relative permittivity variation due to the presence of water [15,16,17]. Moreover, in this case, sources of error are represented by material density, thickness, and the presence of external means (nails, pegs). 

Finally, other methods such as Ground Penetrating Radar (GPR) [18], Nuclear magnetic resonance (NMR) [19], Thermography [20,21], Near-infrared spectrometry (NIRS) [22], and Ultrasound are often used to measure the water in objects, which are stone- or wood-based [23,24,25], but provides its gradient distribution and not punctual information.

As stated above, reflectometry played a leading role in water content measurements, building materials, and woodworks diagnosis in recent years thanks to developing low-cost and tailored probes with adequate measurement accuracy [26]; by way of example, the following works are reported. 

Schajer et al. [27] have proposed a microwaves system based on the measurement of the beam’s attenuation, phase shift, and depolarization, allowing the simultaneous measurement of grain orientation, density, and moisture content.

Aichholzer et al. [12] have proposed a free-space transmission measurement method only in the 8–12 GHz range and based on two opposed linearly polarized horn antennas. The tested samples were moistened in the span of 7.6–14%. In addition, the authors proposed a multiparametric interpolation model for the moisture content/permittivity relationship, also involving the temperature and the operating frequency. 

Razafindratsim et al. [28] have presented a measurement system to evaluate the relative permittivity of three wood species in the broadcast frequency of 1.26 GHz for high moisture content (up to 120%) using the weak perturbation method of the resonant cavity. 

Mai et al. [29] have proposed a comparative assessment to evaluate the relative dielectric permittivity for different moisture conditions, using a GPR instrument with 1.55 GHz antennas and resonance technique at 1.26 GHz. 

Finally, Sahin et al. [30] proposed dielectric measurements at the broadcast frequency of 2.45 GHz and 9.8 GHz for three Euromerican hardwoods, based on the transmission line method.

The permittivity-moisture calibration curves were realized in the last four cases, using the permittivity evaluated at a particular frequency [28,29,30] or the average permittivity over the frequency range [12]. For these reasons, in this paper, we propose a different way to realize permittivity-moisture calibration curves. We first evaluate the simplest parametric fit for the permittivity as a function of frequency and then a frequency-independent calibration curve. The proposed 1–12 GHz frequency range is in line with the literature, but at the same time, no one investigated the whole band, but only particular frequencies (1.26 GHz, 1.56 GHz, 2.56 GHz, 9.8 GHz, or 10 GHz). This range also has many advantages, such as a band suitable for making small sensors or compatible with the passband of low-cost portable instrumentation [31,32].

For these reasons, in this paper, we investigate the possibility of defining a dispersion model for the wood-based object through a low-cost and non-invasive sensor such as an open-ended probe, and evaluating the calibration curves that could be useful for successive applications in the laboratory and on-site, through waveguides. The present work is organized as follows. First, Section 2 describes the chosen materials, the moisturizing and weighing process, and the proposed measurement system based on three different probes: an open-ended probe with a frequency range of 1 to 20 GHz and two rectangular waveguides with a range of 1.72 to 2.60 GHz (namely WR-430) and 8.20–12.40 GHz (namely WR-90). Finally, experimental results are reported in Section 3, while the conclusions and the future work are outlined in Section 4. 

## 2. Materials and Methods

### 2.1. Measurement Setup

As aforementioned in the introductive section, we have used three different probes to characterize the wood samples and evaluate the calibration curve relating permittivity to water content:

- an open-ended coaxial probe (OECP),
- a WR-430 waveguide,
- a WR-90 waveguide.

The choice of three different probes is justified because rectangular waveguides ensure a highly accurate and repeatable setup, which can measure the material permittivity on a relatively small frequency band (smaller than an octave). The two different waveguides were chosen to cover the frequency range around 2 GHz (with the WR-430 system) and 10 GHz (with the WR-90 system). Open-ended coaxial probes, on the contrary, exhibit extremely large operating bandwidth but require incredibly flat and locally homogeneous samples for best accuracy. Furthermore, wood samples show local non-uniformities that make open-ended coaxial measurements scarcely repeatable due to the grain. Nonetheless, open-ended coaxial probe measurements, covering a frequency band that includes the two sub-bands characterized by the two waveguide systems, turned out very useful to investigate a suitable model to describe the dielectric dispersion of wood samples.

**Figure 1:**
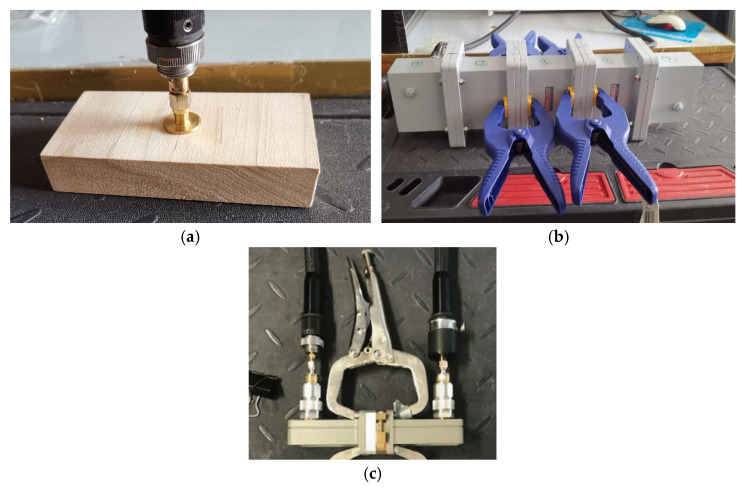
This figure reports (**a**) OEPC, (**b**), WR430, and (**c**) WR90.

All measurements on the wood samples were carried out through an Agilent E8363C vector network analyzer (VNA) equipped with the Agilent 85071E permittivity measurement software, which embeds the NIST model, an algorithm widely used for permittivity retrieval thanks to its high accuracy for nonmagnetic materials. The setups are reported in Figure 1.

The first probe was a customized open-ended coaxial probe, realized from a standard SMA panel connector with an additional flange, connected to the VNA, with a 1–20 GHz bandwidth. This kind of probe is generally used for the permittivity measurements of solids having flat surfaces and liquids. The probe was designed to ensure two essential features: the use in a low-frequency analysis range (below 20 GHz) and has the electromagnetic field penetration depth confined in the samples. To guarantee a suitable compromise between spatial resolution and the capability to detect slight dielectric variations, a probe with 1.25 mm for the outer diameter of the inner conductor and 4.45 mm for the outer diameter of the outer conductor was chosen, with a 0.25 mm annular section. A dielectric section of Teflon insulates the two conductors. The length of the probe is 11 mm, while the flange has a diameter of 17.2 mm. The probe was calibrated through short, air, and acetone measurements.

The second probe was based on a customized WR-430 waveguide system, operating at 1.7–2.6 GHz [33]. The system is characterized by a sample holder of 109 × 55 × 100 mm connected to the VNA. Finally, the last probe is based on a commercial WR-90 waveguide system, operating at 8.2–12.4 GHz. The system is characterized by a sample holder of 22.8 × 10.1 × 8.2 mm connected to the VNA. Both waveguides were calibrated with a TRL calibration kit.

### 2.2. Wood Sample

The measurements were conducted on four different wood species:

- Fir (*Abies alba Mill., 1759*),
- Poplar (*Populus alba* L. *1753*),
- Beech (*Fagus sylvatica* L., *1753*),
- Oak (*Quercus petraea (Matt.) Liebl*).

**Figure 2:**
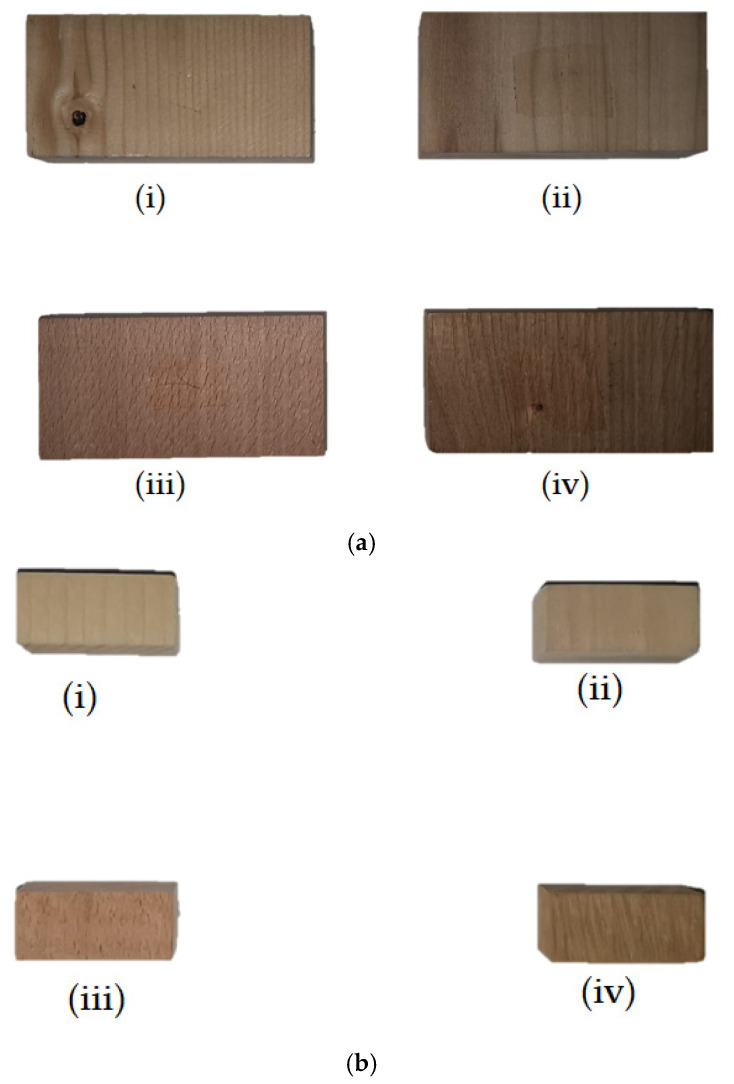
(**a**) Samples for WR-430 and (**b**) samples for WR-90. In each figure, (i) fir, (ii) poplar, (iii) beech, and (iv) oak wood.

Fir and beech wood were widely used in eastern Europe for the construction of roofs and ceilings [34] and musical instruments [35], while poplar and oak wood found wide use as support in painting in Italy and Germany, respectively [36,37]. For each type of wood, two samples were realized as follows and reported in Figure 2:

- Four samples were cut to be inserted in the WR-430 waveguide with a length L = 109.2 mm, a width W = 54 mm, and a thickness T = 15 mm. All samples are cut along the same grain line.
- Four samples were cut to be inserted in the WR-90 waveguide with a length l = 22.2 mm, a width w = 10 mm, and a thickness t = 8 mm. All samples are cut along the same grains line and the grain of the larger samples.

### 2.3. Moisturing and Weighing Procedure

**Scheme 1:**
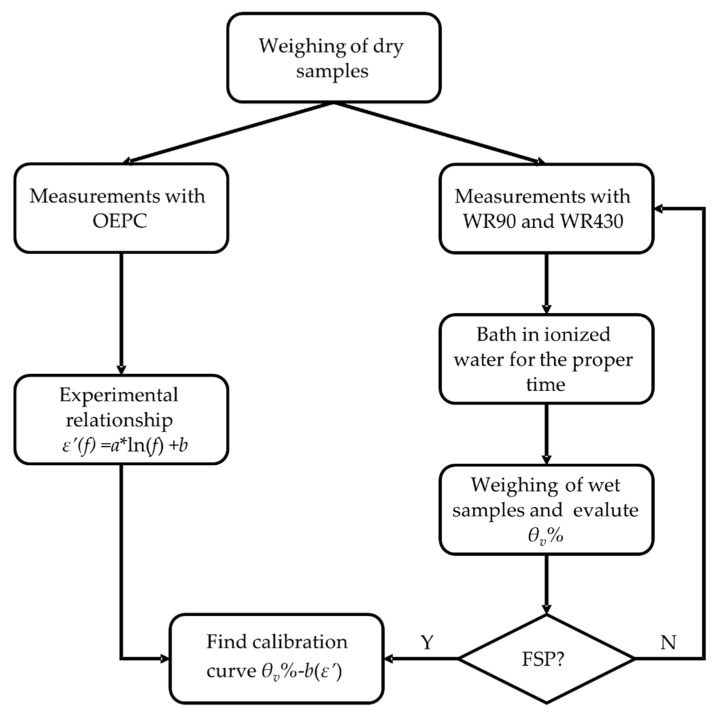
The scheme reports measurement workflow and highlights the combination of the two setups to determine the calibration curves.

The realized samples were preliminarily dried in a ventilated oven at (105 ± 5) °C until the sample weight had become stable for three consecutive measurements. After this process, the sample was bathed in deionized water at least seven times, immersing the sample for 90 s for WR-430 samples and 35 s for WR-90 samples, until different water content levels were obtained in the 0–5% span. The measurement process was described in Scheme 1.

The water content percentage (*WC*_%_) was calculated according to EN 16682-2017 [38] and shown in Equation (1).

(1) $$C_{\%}=\frac{W_{i}-W_{d}}{V\cdot \rho_{H_{2}O}}$$

with *W_d_* the dried weight, *W_i_* the *i*-th water content level, *V* the volume of the sample and $\rho_{H_{2}O}$ the deionized water density (0.998 mg/mm^3^); all parameters are expressed in grams. The weight has been acquired through a 10 mg resolution electronic balance before and after measurements to evaluate the evaporation effect. The water content was assessed as the average value.

## 3. Results and Discussions

### 3.1. OECP Results

**Figure 3:**
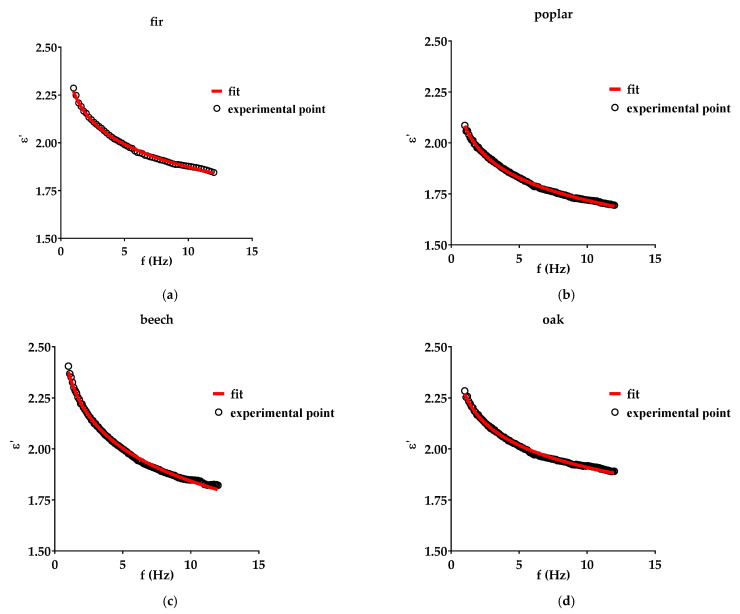
This figure reports the relations between the real part of permittivity *ε*′ and frequency *f* at the dry condition with highlighted in red the log-fit: (**a**) fir *ε*′–*f* relationship; (**b**) poplar *ε*′–*f* relationship; (**c**) beech *ε*′–*f* relationship; (**d**) oak *ε*′–*f* relationship. Measurements were conducted with a customized open-ended probe.

The measurement results obtained with the OECP for each wood sample are shown in Figure 3a–d. In particular, Figure 3a shows the fir results, while poplar, beech, and oak results are presented in Figure 3b–d, respectively. Each figure shows the measured real part of dielectric permittivity *ε*′ as a function of the frequency *f*, at the dry condition. 

The (*ε*′–*f)* curves fittings were evaluated through a non-linear least-squares fit to identify the mathematical relationship of *ε*′(*f*). The resulting fitting equation was for: (i) fir sample $\varepsilon_{f}^{\prime }=-0.54\ln\left(f\right)+2.38$ with a coefficient of determination *R^2^* = 0.996, (ii) poplar sample $\varepsilon_{p}^{\prime }=-0.54\ln\left(f\right)+3.16$ with an *R^2^* = 0.996, (iii) beech sample $\varepsilon_{b}^{\prime }=-0.46\ln\left(f\right)+3.65$ with an *R^2^* = 0.995, and (iv) oak sample $\varepsilon_{o}^{\prime }=-0.54\ln\left(f\right)+3.70$ with an *R^2^* = 0.992. 

### 3.2. Waveguide Results

**Figure 4:**
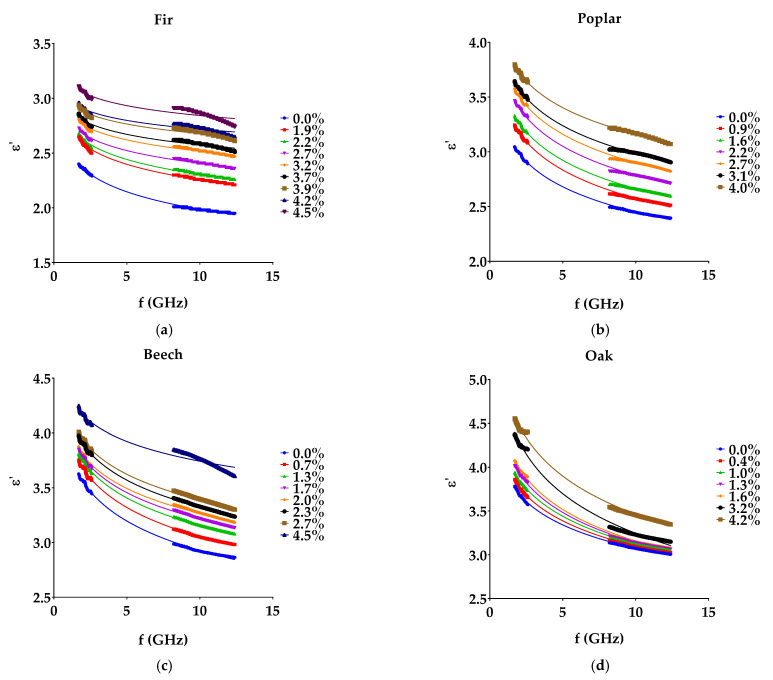
This figure reports the relations between *ε*′ and frequency *f* for the different water content θ_v_ with highlighted the log-fit: (**a**) fir *ε*′–*f* relationship; (**b**) poplar *ε*′–*f* relationship; (**c**) beech *ε*′–*f* relationship; (**d**) oak *ε*′–*f* relationship. Measurements were conducted with WR-430 and WR-90 waveguides.

Starting with the fitting model evaluated from the OECP results, the log-fit was used to fill the frequency gap between the measurements obtained at different water contents with the WR-430 and WR-90 waveguide probes. The measurement results obtained with the WR-430 and WR-90 systems are presented in Figure 4a–d.

First, Figure 4a reports the *fir* (*ε*′–*f*) plot as a function of the water content variation, in the range of 0.0% to 4.5%. Then, Figure 4b reports the *poplar* (*ε*′–*f*) plot as a function of the water content variation, in the range of 0.0% to 4.0%. Next, Figure 4c reports the *beech* (*ε*′–*f*) plot as a function of the water content variation, in the range of 0.0% to 4.5%. Finally, Figure 4d reports the *oak* (*ε*′–*f*) plot as a function of the water content variation, in the range of 0.0% to 4.2%. Using the results obtained with OECP for each wood sample, it is possible to observe how the log-fit interpolation based on the equation y=aln(x)+b still describes the (*ε*′–*f*) relationship as the water content in the samples increased, with an *R^2^* > 0.93. For the higher water contents, the fitting at the higher frequencies shows a poorer performance because the dielectric relaxation of water becomes apparent. The results for each sample are presented in Table 1.

### 3.3. Calibration Results

**Figure 5:**
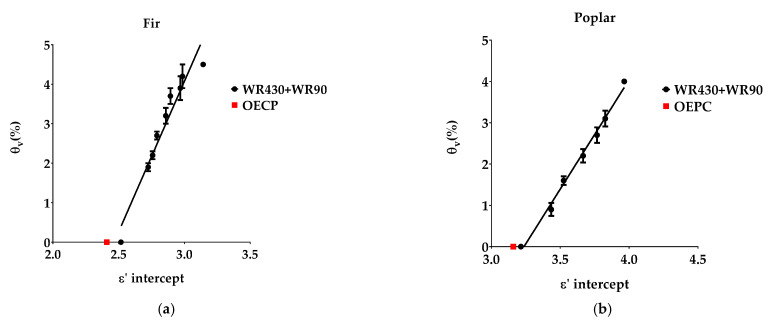

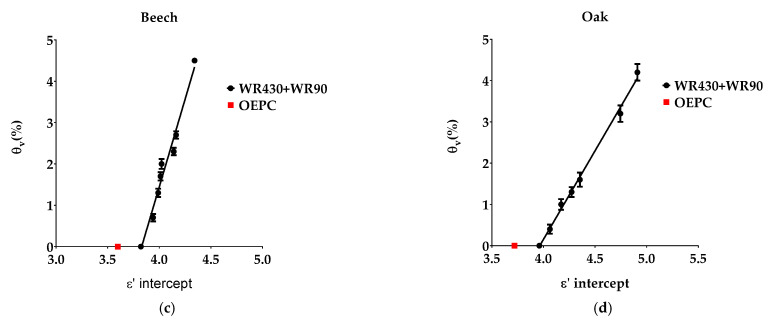
This figure reports the calibration curve based on the relationship between the vertical shift *b* of the log-fit equation and the different *θv (%)* levels: (**a**) fir, (**b**) poplar, (**c**) beech, (**d**) oak. Measurements were conducted with WR-430 and WR-90 waveguides. In red, the OEPC measurements at dry condition were highlighted.

Table 1 above highlights how a water content increase corresponds to an increase in the b parameters for each wood. In Figure 5a–c, the calibration curves of water content are reported as a function of the vertical shift *b* (*ε*′). For the fir sample, the calibration curve is $\theta_{v}\%=7.61\cdot b-17.78$ with an *R^2^* = 0.96, while the poplar sample has a calibration curve $\theta_{v}\%=5.29\cdot b-17.14$ with an *R^2^* = 0.99. Similarly, beech and oak samples have a calibration curve $\theta_{v}\%=8.48\cdot b-32.46$ with *R^2^* = 0.97 and $\theta_{v}\%=4.29\cdot b-17.00$ with *R^2^* = 0.99, respectively. The *R^2^* was consistently higher than 0.95, highlighting the method potentiality to linearly correlate the water content with the vertical shift in experimental data. Moreover, by the comparison between the EOCP and waveguide results at the dry condition, it is possible to see how the vertical shift for the two configurations has a difference always lower than 7.0% (3.1% for poplar sample, 5.7% for the fir and beech samples and 6.6% for oak). These differences depend on the method applied because OEPC performs measurement in a small volume and a thin section near the probe and is therefore influenced by the local grain, while the WR systems do a measurement through the entire volume of samples.

## 4. Conclusions and Prospects

This work presented a combined reflectometric setup based on an Open-Ended Coaxial Probe and two waveguides (WR-430 and WR-90) to evaluate the calibration curve relating water to permittivity for four different wood species.

The investigated calibration curve exploits the fact that an increase in the water content corresponds to an increasing dielectric permittivity. For example, a dry wood sample has a permittivity *ε_r_* in the range of 2–3 while water presents an *ε_r_* = 80. The results obtained by OECP on dry sampled permitted the evaluation of the proper fit model over frequency for wood, showing an *R^2^* always higher than 0.995. Furthermore, the subsequent application of the log-fit model to the data obtained with the waveguides showed how this model adequately fits them when there is an increase in the water content. For these measurements, the *R^2^* is higher than 0.95. Finally, a linear calibration curve relating the parameters of the frequency dispersion model to water content was obtained with a coefficient of determination higher than 0.96.

Furthermore, the results obtained with OEPC suggest that this method could be used directly in on-site applications showing a difference with a more accurate method lower than 7%. These results will be used to design innovative and small-size probes (resonant patch sensor, OPEC, etc.) for non-invasive water content measurement, with the possibility of being glued directly on the surfaces to be monitored and the aim of a medium-long period of observation. Moreover, the investigation of the calibration curve of new wood species will be carried out.

## Figures and Tables

**Table 1:** Log-fit parameters and coefficient of determination for the wood samples at different levels of water content.

Parameter	Fir ^1^								
*θ_v_ (%)*	0.0%	1.9%	2.2%	2.7%	3.2%	3.7%	3.9%	4.2%	4.5%
*a*	−0.53	−0.47	−0.45	−0.38	−0.34	−0.32	-0.29	−0.27	−0.30
*b*	2.52	2.73	2.76	2.79	2.86	2.89	2.969	2.986	3.14
*R^2^*	0.999	0.998	0.991	0.986	0.980	0.975	0.958	0.941	0.932
**Parameter**	**Poplar** **^1^**								
*θ_v_ (%)*	0.0%	0.9%	1.6%	2.2%	2.7%	3.1%	4.0%		
*a*	−0.77	−0.86	−0.86	−0.88	−0.87	−0.84	−0.80		
*b*	3.26	3.44	3.53	3.67	3.77	3.83	3.97		
*R^2^*	0.999	0.999	0.999	0.998	0.998	0.998	0.998		
**Parameter**	**Beech** **^1^**								
*θ_v_ (%)*	0.0%	0.7%	1.3%	1.7%	2.0%	2.3%	2.7%	4.5%	
*a*	−0.90	−0.89	−0.83	−0.79	−0.76	−0.81	−0.77	−0.60	
*b*	3.82	3.94	3.99	4.01	4.02	4.14	4.16	4.34	
*R^2^*	0.999	0.998	0.998	0.998	0.996	0.998	0.997	0.978	
**Parameter**	**Oak ^1^**								
*θ_v_ (%)*	0.0%	0.4%	1.0%	1.3%	1.6%	3.2%	4.2%		
*a*	−0.89	−0.97	−1.05	−1.13	−1.20	−1.50	−1.45		
*b*	3.96	4.06	4.17	4.27	4.35	4.75	4.91		
*R^2^*	0.998	0.998	0.999	0.998	0.998	0.995	0.995		

^1^ For each wood sample and water contents, the log-fit equation is y=aln(x)+b.

## Data Availability

Not applicable.
